# Incarcerated Gravid Uterus in a Nulliparous Female Presenting With Urinary Hesitancy and Rectal Pressure: A Case Report

**DOI:** 10.7759/cureus.41289

**Published:** 2023-07-02

**Authors:** Christine L Prochnow, Megan L Hilbert, Christopher R Bickett, Jeremy S Boyd

**Affiliations:** 1 Emergency Medicine, Vanderbilt University Medical Center, Nashville, USA; 2 Emergency Medicine, University of Pittsburgh Medical Center, Pittsburgh, USA; 3 Emergency Medicine, Veterans Affairs Tennessee Valley Health Services, Nashville, USA

**Keywords:** obstetrics-gynecology, emergency medicine, point-of-care ultrasound, pregnancy, rectal pressure, urinary retention, retroverted uterus, incarcerated uterus

## Abstract

Incarcerated gravid uterus (IGU) is a rare condition that is associated with urinary obstruction, sepsis, peritonitis, and ultimately maternal death. IGU occurs when the retroverted uterus in a gravid patient becomes trapped in the pelvis during the second trimester. We present the case of a nulliparous female who came to our emergency department (ED) at 14 weeks and five days gestation with new onset intermittent urinary hesitancy and rectal pressure starting approximately 10 days prior to presentation. IGU was diagnosed based on pelvic examination and ultrasound in the ED. Emergency physicians should have a high index of suspicion for IGU in their differential diagnosis for pregnant females with urinary and rectal complaints. Point-of-care ultrasound (POCUS) should be used as an adjunct in identifying this condition.

## Introduction

Incarcerated gravid uterus (IGU) is a rare condition where the retroverted uterus becomes trapped between the sacral promontory and the pubic symphysis and does not spontaneously reduce as the pregnancy progresses [1]. While uterine retroversion is recognized as a normal anatomical variant in first-trimester pregnancy, with some studies suggesting it occurs in 15-25% of early pregnancy, the gravid uterus typically grows into the abdominal cavity from the pelvis after the 14th week of gestation [2, 3]. If the gravid uterus becomes incarcerated, the continued fetal growth will cause the cervix to displace superiorly, pushing against the pubic symphysis and subsequently compressing the urethra and bladder, causing bladder outlet obstruction. Known risk factors for the development of IGU include adhesions from prior surgery, pelvic inflammatory disease, endometriosis, large uterine fibroids, uterine malformations, and prior uterine incarceration [1, 3, 4]. While the literature primarily consists of IGU in case reports, documented complications include urinary tract obstruction, urinary tract infection, renal failure, thrombosis, fetal growth restriction, fetal demise, and uterine wall necrosis/rupture [1, 3-5]. Emergency department (ED) diagnosis of IGU is rare, and medical literature describing these cases with point-of-care ultrasound (POCUS) in the ED is even rarer. We report a case of IGU diagnosed in the early second trimester in our ED, review the clinical diagnostic criteria of IGU, findings supportive of IGU on POCUS in addition to imaging pitfalls, and discuss the implications for emergency medicine providers. 

## Case presentation

**Figure 1:**
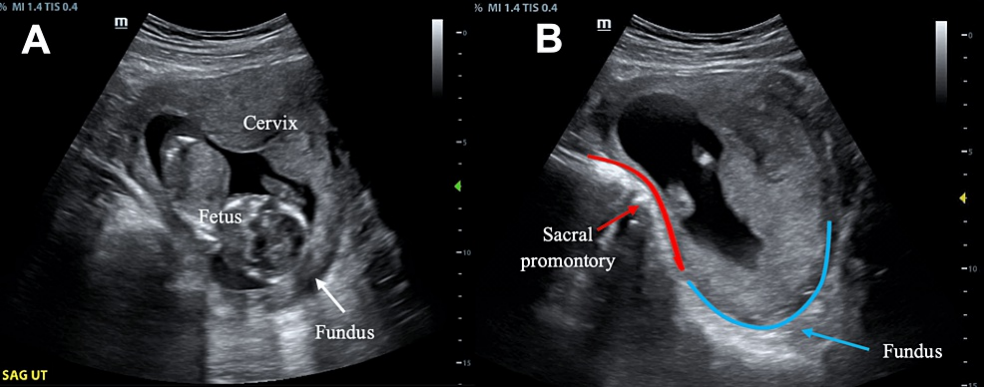
Point-of-care transabdominal ultrasound images of incarcerated gravid uterus in the sagittal plane. A) Sagittal image demonstrates the superiorly displaced cervix and lower uterine segment above the uterine fundus. B) Parasagittal image shows the uterus is fixed in retroversion with the fundus (marked by the blue line) trapped below the sacral promontory (marked by the red line) seen while fanning the ultrasound transducer through the sagittal and parasagittal planes.

A 31-year-old female, gravida 2, para 0010, presented to our ED at 14 weeks and five days of gestation with chief complaints of intermittent but progressively worsening urinary hesitancy, the sensation of incomplete voiding, suprapubic discomfort, and rectal pressure for approximately 10 days. She had no systemic infectious symptoms or vaginal bleeding. She reported a past medical history significant for endometriosis with prior endometrioma resection and a miscarriage 10 years prior to this pregnancy. The patient had her first ultrasound for the current pregnancy 12 days prior to presentation, which confirmed a single viable intrauterine pregnancy. Upon presentation, her initial blood pressure was 145/78, which improved to 118/65 without intervention. Her heart rate was 86 beats per minute, respiratory rate 18, and oxygen saturation 100% on room air with a temperature of 98F. The patient appeared well. Her abdominal exam was non-tender with palpation, but the patient did have subjective worsening urinary urgency with palpation of the lower abdomen. The pelvic exam was notable for the inability to visualize the cervix on the speculum exam, and the bimanual exam revealed a closed, anterior cervix without cervical motion tenderness or palpable masses. There was no evidence of pelvic organ or rectal prolapse. Urinalysis was without evidence of a urinary tract infection. IGU was diagnosed based on the pelvic exam in conjunction with ultrasound, which demonstrated a retroverted uterus with superior displacement of the cervix (Figure 1, Video 1). Appropriate fetal movement and heart rate were also noted.

**Video 1:** Incarcerated gravid uterus

**Figure 2:**
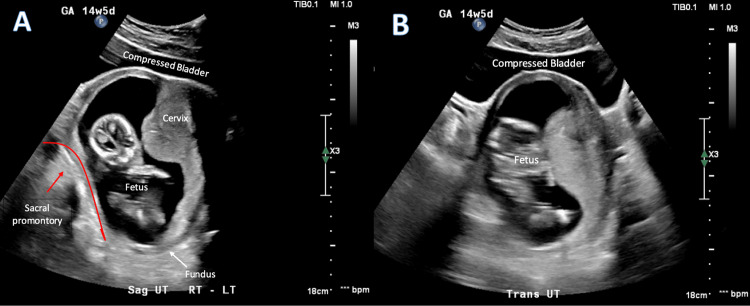
Comprehensive transabdominal ultrasound images of incarcerated gravid uterus in the sagittal and transverse plane A) Sagittal image demonstrates the superiorly displaced cervix and lower uterine segment above the uterine fundus, with superior displacement of the urinary bladder and resultant bladder outlet obstruction. The fundus is trapped below the sacral promontory (marked by the red line). B) Transverse image redemonstrates bladder outlet obstruction due to the upward compression of the displaced uterus on the urinary bladder.

The patient was initially able to void in order to provide a urine sample on arrival to the ED, with a decompressed bladder noted on transabdominal POCUS. Imaging of the bladder outlet obstruction characteristically seen with IGU was captured shortly afterward when a comprehensive ultrasound was ordered in the ED (Figure 2). Upon returning from a comprehensive ultrasound, the patient notified her ED nurse that she could no longer void despite feeling the need to urinate with associated urinary urgency. Therefore, a urinary catheter was placed for the patient's urinary retention while undergoing evaluation. It is unclear what degree of urinary retention the patient was experiencing, as the volume of output upon placement of the urinary catheter was not reported. 

The suspected etiology of bladder outlet obstruction and, ultimately, her urinary retention were thought to be secondary to cervical displacement, resulting in compression of the urethra. As such, obstetrics was consulted, and the patient was taken to labor and delivery for attempted bedside manual reduction of the uterus. Per the electronic medical record, bedside manual reduction was attempted by obstetrics with IV fentanyl and midazolam provided by the anesthesia team. Due to discomfort and inability to tolerate bedside manual reduction, the patient was taken to the operating room for monitored anesthesia care.

In the operating room, the urinary catheter remained in place, and the patient was placed in the dorsal lithotomy position. Adequate sedation was achieved with IV propofol. Successful reduction was achieved on the second intra-operative attempt by increasing flexion of the hips with manual pressure applied intravaginally to the palpable fundus in the cephalad direction, enabling the uterine fundus to be lifted out of the pelvis. After reduction, the uterus was palpable within the abdomen. Repeat POCUS by the obstetrics team confirmed appropriate fetal heart tones after successful reduction per the operative note. However, the POCUS images were not saved in our picture archiving and communication system for later review. The patient went to the operating room thirteen hours after her initial ED presentation, and she was observed for two hours postprocedure, after which time she was discharged home.

The pregnancy was ultimately complicated by anemia with a hematocrit of 28%, an episode of nausea and vomiting with preterm uterine contractions at 33 weeks of gestation that resolved after intravenous hydration. When the patient was later admitted for planned induction at 40 weeks of gestation, fetal monitoring showed late fetal heart rate decelerations with concern for placental abruption. The patient was taken to the operating room, where a primary lower segment transverse cesarean section was performed that demonstrated a 10% placental abruption. The delivery of the infant was otherwise without complication, and both the infant and mother recovered uneventfully. 

## Discussion

Pregnant patients commonly present to the ED with urinary complaints and abdominal discomfort. IGU should be considered in the differential diagnosis. Among cases of IGU in the literature, the majority present in the second trimester with nonspecific complaints, including dysuria, urgency, frequency, urinary retention, abdominal and pelvic pain, constipation, back pain, perineal pain, tenesmus, and a painful mass prolapsed outside the anus [1]. In a literature review performed by Han et al. of 162 cases of IGU, the most common symptoms are urinary manifestations (53.70%) due to the resultant bladder outlet obstruction caused by the displacement of the cervix with 51.88% of IGU cases diagnosed between 13 to 26 weeks gestation [1]. IGU is a rare diagnosis occurring in only one in 3000 to 10,000 pregnancies. While a rare diagnosis, emergency medicine providers should have increased clinical suspicion for pregnant patients presenting with urinary and/or rectal complaints [2-4]. With the rising utilization of in vitro fertilization (IVF) and overlapping risk factors for IGU and infertility, there is the potential possibility that the frequency of IGU may also increase. We found two cases (one case report and one journal correspondence within the past five years) documenting IGU associated with IVF [1]. 

The clinical diagnostic criteria for IGU are based on symptoms and exam: pain and difficulty voiding along with a palpable mass in the cul-de-sac due to severe anterior displacement of the cervix behind the pubic symphysis. Definitive diagnosis is made using transabdominal ultrasound or magnetic resonance imaging (MRI) in conjunction with the clinical presentation. Given the rising utilization of POCUS in emergency medicine as well as its immediate accessibility and relatively low cost, transabdominal ultrasound imaging should be performed first. The POCUS operator should focus on the key anatomic relationships of the pelvis, including the position of the lower uterine segment and cervix relative to the fundus. If the diagnosis remains unclear, then proceed with a comprehensive ultrasound or MRI if available.

A total of seven papers were identified in the emergency medicine literature published from 1986-2022, containing a total of 10 case reports with IGU diagnosed in the ED. Of these case reports, there are only two published corresponding images demonstrating findings consistent with IGU. This case report improves on the previously published images by demonstrating clearly the relationship between the retroverted uterine fundus trapped below the sacral promontory as well as the anteriorly displaced cervix [5-6]. Ultrasound in the setting of IGU will typically demonstrate a fluid-filled, distended bladder; however, ours did not because the patient had successfully voided before our imaging was performed. Imaging of bladder outlet obstruction was captured shortly afterward when a comprehensive ultrasound was ordered in the ED - by this time, the patient had developed acute urinary retention requiring urinary catheter placement.

Transabdominal ultrasound and especially MRI have the advantage of providing improved visualization of the anatomic relationships in the pelvis, which is essential for the diagnosis of IGU [7]. This is in contrast to transvaginal ultrasound, which is less helpful in identifying anatomic relationships and can lead to misinterpretations. Initial suspicion for IGU should increase when the cervix cannot be easily identified on ultrasound, as occurred in this patient's case. As the cervix becomes displaced by the IGU, typical anatomical relationships seen on ultrasound are no longer present. Typically, the vaginal stripe and cervix are inferior to the bladder, with a downward slope dorsally to meet with the uterus in the sagittal plane. However, with IGU, the fundus will be seen posteriorly below the sacral promontory, and the cervix extends upward, superior to the uterus, and becomes obscured or elongated, while the bladder is displaced superiorly. The compression caused by the cervix, as well as the enlarging uterus, leads to urinary retention with a distended and elongated bladder seen on POCUS, which is seen in Figure 2 for comparison. Emergency medicine physician familiarity with the typical anatomic relationships may also help screen for IGU, allowing for even earlier identification and referral in asymptomatic patients with IGU presenting earlier in gestation for other complaints. 

Physicians should readily discuss images with their institution's obstetrician and radiologist if they have concerns about IGU. Misinterpretations documented in the literature include leiomyoma/suspected leiomyoma necrosis, ectopic pregnancy, abdominal pregnancy, and placenta previa [7-9]. As mentioned previously, uterine retroversion is reported in 15-25% of pregnancies; however, there is limited data regarding the percentage that spontaneously resolves. A retrospective study performed at a center in Japan identified 14 IGU cases out of 6958 pregnant women over the course of 10 years, where IGU was defined as failure of the uterine fundus to self-correct after 16 weeks of gestation [9]. Among the 14 cases, 11 had spontaneous resolution of IGU between 16-31 weeks gestation; however, the paper does not discuss any possible clinical complications of expectant management in the third trimester, with the exception of an IGU that was misdiagnosed as placenta previa resulting in a cervical laceration during cesarean delivery. Thirteen of these women had gynecological risk factors for IGU. While our patient had the known risk factor of endometriosis, her operative report did not mention adhesions or other uterine malformations that would prevent the uterus from entering the abdominal cavity.

Managing patients with IGU is controversial since the literature on IGU consists primarily of case reports and literature reviews. There are no specific published guidelines for the management of IGU, and therefore optimal management is debated given the lack of high-quality evidence. Options for management vary and are typically recommended from least invasive with progression to more invasive methods depending on clinical course. Such options include expectant management while in the first trimester (if asymptomatic), passive reduction with the knee-chest position, or manual reduction with both maneuvers performed only after emptying the bladder with a urinary catheter. More invasive options include colonoscopy or surgery, which are indicated when conservative measures fail. The decision to proceed with colonoscopy versus surgery is beyond the scope of this case report and should be determined by an obstetrician after a risk/benefits discussion with the patient. However, a colonoscopy is less invasive than a laparotomy, and there are multiple case reports of successful reduction. 

The current consensus is for the obstetrician to attempt manual reduction between 14-20 weeks, with the caveat that attempts later than 20 weeks are likely to fail and increase the potential for preterm labor [10]. Our patient was symptomatic at 14 weeks and five days of gestation with the successful manual reduction in the operating room after urinary catheter placement. Given that no clear treatment guidelines exist, current recommendations are weak in the setting of limited data. We recommend prompt consultation with an obstetrician when IGU is diagnosed in the ED, as there is significant potential for severe complications. If the patient is experiencing urinary symptoms, placing a urinary catheter to prevent bladder outlet obstruction and resultant complications such as kidney injury is a reasonable first step in management by an emergency medicine physician while awaiting recommendations from an obstetrician. 

## Conclusions

While a rare diagnosis, emergency medicine physicians should consider IGU in pregnant females presenting with urinary, abdominal, and rectal complaints. In addition, the complaint of urinary retention in a pregnant female should significantly increase the clinician's index of suspicion for IGU. Definitive diagnosis can be made when point-of-care ultrasound demonstrates the cervix pulled anterior to the uterus in conjunction with a history of urinary and/or rectal symptoms with the inability to visualize the cervix and a palpable mass in the cul-de-sac on the exam. These findings should prompt the treating physician to consult an obstetrician and expedite management to prevent maternal and fetal complications.
